# Surveillance of Arrhythmia in Patients After Myocardial Infarction Using Wearable Electrocardiogram Patch Devices: Prospective Cohort Study

**DOI:** 10.2196/35615

**Published:** 2022-06-09

**Authors:** Ju-Seung Kwun, Chang-Hwan Yoon, Sun-Hwa Kim, Ki-Hyun Jeon, Si-Hyuck Kang, Wonjae Lee, Tae-Jin Youn, In-Ho Chae

**Affiliations:** 1 Seoul National University Bundang Hospital Seongnam-Si Republic of Korea

**Keywords:** myocardial infarction, arrhythmia, wearable electronic device, wearable, ECG, electrocardiogram, patch, patch devices, atrial fibrillation, heart, rhythm, cardiology, cardiologist, cohort study, tachycardia, beta-blocker

## Abstract

**Background:**

Acute myocardial infarction may be associated with new-onset arrhythmias. Patients with myocardial infarction may manifest serious arrhythmias such as ventricular tachyarrhythmias or atrial fibrillation. Frequent, prolonged electrocardiogram (ECG) monitoring can prevent devastating outcomes caused by these arrhythmias.

**Objective:**

We aimed to investigate the incidence of arrhythmias in patients following myocardial infarction using a patch-type device—AT-Patch (ATP-C120; ATsens).

**Methods:**

This study is a nonrandomized, single-center, prospective cohort study. We evaluated 71 patients who had had a myocardial infarction and had been admitted to our hospital. The ATP-C120 device was attached to the patient for 11 days and analyzed by 2 cardiologists for new-onset arrhythmic events.

**Results:**

One participant was concordantly diagnosed with atrial fibrillation. The cardiologists diagnosed atrial premature beats in 65 (92%) and 60 (85%) of 71 participants, and ventricular premature beats in 38 (54%) and 44 (62%) participants, respectively. Interestingly, 40 (56%) patients showed less than 2 minutes of sustained paroxysmal atrial tachycardia confirmed by both cardiologists. Among participants with atrial tachycardia, the use of β-blockers was significantly lower compared with patients without tachycardia (70% vs 90%, *P*=.04). However, different dosages of β-blockers did not make a significant difference.

**Conclusions:**

Wearable ECG monitoring patch devices are easy to apply and can correlate symptoms and ECG rhythm disturbances in patients following myocardial infarction. Further study is necessary regarding clinical implications and appropriate therapies for arrhythmias detected early after myocardial infarction to prevent adverse outcomes.

## Introduction

Acute myocardial infarction is a common cardiac emergency associated with high potential for mortality and a substantial risk of complications [1]. The majority of patients with acute myocardial infarction develop some form of arrhythmia during or immediately after the events, and more than 10% of these patients manifest serious arrhythmias such as ventricular tachyarrhythmias or atrial fibrillation, which may cause disabling stroke and sudden cardiac death [2,3]. These adverse events most frequently occur during the first months after myocardial infarction [4]. Therefore, close electrocardiographic (ECG) monitoring during this period is extremely important to avoid severe outcomes.

However, conventional ECG monitoring devices such as multilead portable ECG monitoring or Holter monitoring devices are not useful for continuous monitoring of ECG signals for longer than 24 hours, particularly after discharge from the hospital [5]. In addition, implantable loop recorders allow for a longer duration of ECG monitoring [6] but require an invasive procedure, making patients vulnerable to infection and discomfort. Due to these technical and other difficulties, data regarding types and frequencies of arrhythmia after acute myocardial infarction are scarce.

To compensate for these drawbacks, a new generation of ECG monitoring devices with advanced technologies have been developed [7]. The Zio Patch (iRhythm Technologies), a single-use, patch-type continuous ECG monitoring device, can continuously monitor the patient’s ECG signals for 2 weeks and has been applied to more than 400,000 patients [8]. This device enables a longer duration of monitoring, as well as wireless data transfer and unlike conventional ECG monitoring devices, does not interrupt the daily life of patients [9].

A wearable ECG monitoring patch device can detect ECG rhythm disturbances in patients with postmyocardial infarction. In this study, we investigated the incidence of arrhythmias in patients with postmyocardial infarction using another new wearable patch-type device—AT-Patch (ATP-C120; ATsens).

## Methods

### Recruitment

This study is a nonrandomized, single-center, prospective cohort study. We evaluated patients who had been admitted to our hospital for myocardial infarction and discharged after treatment. Eligible patients had a history of acute myocardial infarction and provided written informed consent to participate. Exclusion criteria were as follows: (1) previously diagnosed with atrial fibrillation, (2) implanted pacemaker, cardioverter-defibrillator, or any electrical devices, and (3) skin problems such as allergic contact dermatitis. Our prospective study enrolled 73 adults aged 55 years or older from April 2020 to November 2020. Among them, 2 participants dropped out due to loss of the device before data acquisition.

### The Experimental Wearable Patch-Type Device

**Figure 1:**
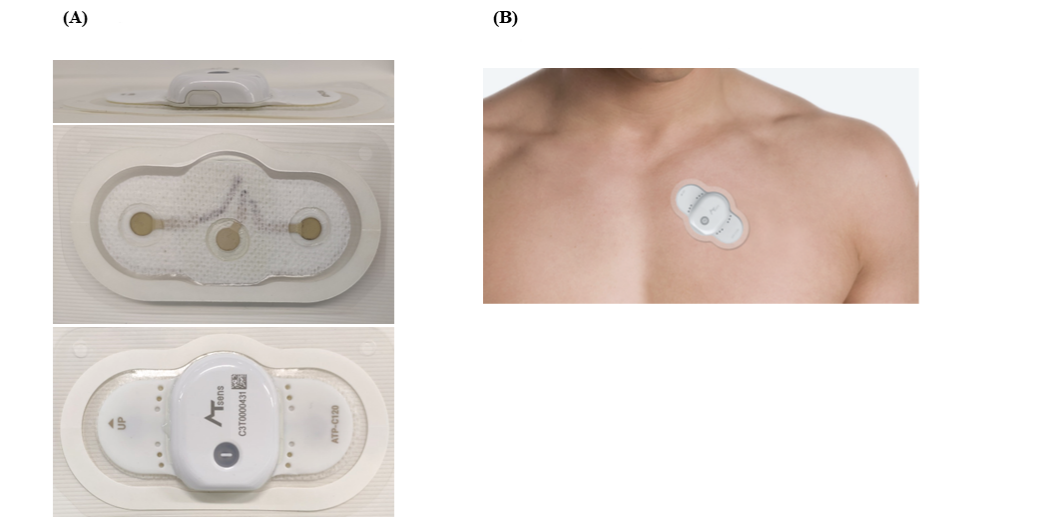
The appearance of ATP-C120 (A) The actual photographs of the ATP-C120 (image courtesy of ATsens). (B) The placement of the ATP-C120 patch (image courtesy of ATsens).

ATP-C120 is a single-lead ECG monitoring device that can continuously monitor the ECG signals for as long as 14 days (11 days if the device is connected to a smartphone via Bluetooth) when attached to the skin over the area of the heart (Figure 1). The device weighs about 13 g, with dimensions of 95.0 × 50.6 × 8.3 mm. This is the smallest of the contemporary wearable patch devices worldwide.

When the device is attached to the patient, several predefined methods are used to prevent the occurrence of noise or signal loss. First, the skin is cleansed and disinfected using a 70% ethanol solution. Skin hair is removed if necessary. Subsequently, the protective film is removed from the patient-side surface of the device. The device is placed at the left third intercostal space, tilted inward 45 degrees. A continuous ECG signal is recorded to a memory card for 11 days. Subsequently, the device is linked to a computer and the data are downloaded and analyzed by a specific program (AT-report) provided by ATsens.

### Trial Schedule

According to institutional Good Clinical Practice for medical devices, we attached the experimental ATP-C120 device to participating patients. We obtained demographic data, as well as past and present medical and drug administration history, and conducted physical examinations (height, body weight, and vital signs such as systolic and diastolic blood pressure and pulse). In addition, laboratory parameters (complete blood count, electrolyte, blood urea nitrogen, creatinine, estimated glomerular filtration rate, liver function tests, lipid panel, fasting blood sugar, and glycated hemoglobin A_1c_ or HbA_1c_) and the 12-lead ECG results before the attachment of the ATP-C120 device were obtained. We detached the device after 11 (SD 5) days and recorded the date and time. Two independent cardiologists analyzed the recorded data for arrhythmic detection.

### Sample Size Calculation

We hypothesized that ATP-C120 could detect 10% of new-onset atrial fibrillation in patients with postmyocardial infarction. We set the type I error as .05 and confidence limit as 5%. Application of these parameters resulted in 139 participants. The attrition rate was set as 5% and, thus, the final sample size was 146 participants. However, we only enrolled 73 patients because of limited enrollment time and funding, which was adjusted by the project manager of the Korean Health Industry Development Institute.

### Statistical Analyses

Data are presented as numbers and frequencies for categorical variables and as mean (SD) for continuous variables. The incidence of atrial fibrillation followed by myocardial infarction is described as proportions and 95% CIs. A 2-sided *P* value of <.05 was indicative of a statistically significant difference. To evaluate interobserver reliability and interdevice reliability, Cohen κ coefficient was calculated. For comparisons between patients with or without atrial tachycardia, chi-square test (or Fisher exact test when any expected count was <5 for a 2 × 2 table) was performed for categorical variables. Statistical analyses were performed using R (version 3.1.0; The R Foundation for Statistical Computing).

### Ethics Approval

The study was reviewed and approved by the Institutional Review Board of Seoul National University Bundang Hospital (B-2003/603-002).

## Results

### Baseline Characteristics

A total of 71 patients who had a history of acute myocardial infarction were included in the analyses. The baseline characteristics are shown in Table 1. The mean age was 67.6 (SD 8.3) years, and 59 (83%) patients were men. Among the study population, 23 (32%) patients were clinically diagnosed with ST-segment elevation myocardial infarction, and 40 (56%) participants were attached with the ATP-C120 device within 6 months of acute myocardial infarction. Patients with previous heart failure were not enrolled in this study. The average left ventricular ejection fraction (LVEF) was 57.1% (SD 8.2%), and pro–B-type natriuretic peptide level was 370.3 (SD 505.3) pg/L. For medication, 61 (86%) participants received aspirin, whereas 37 (52%) participants received P2Y12 inhibitor. In addition, among the study population, 56 (79%) patients received β-blockers, 23 (32%) patients received renin-angiotensin system inhibitors, and 18 (25%) patients received calcium channel blockers.

**Table 1:** Profile of study population (N=71).

Characteristics			Values
Age (years), mean (SD)			67.6 (8.3)
**Gender, n (%)**			
	Male	59 (83)	
	Female	12 (17)	
Systolic blood pressure (mm Hg), mean (SD)			129.8 (18.6)
Diastolic blood pressure (mm Hg), mean (SD)			73.5 (11.3)
Heart rate (beats/min), mean (SD)			71.6 (11.8)
BMI (kg/m^2^), mean (SD)			24.3 (3.0)
Clinical diagnosis of STEMI^a^, mean (SD)			23 (32)
**Post** **myocardial infarction** **period, n (%)**			
	<6 months	40 (56)	
	6-12 months	3 (4)	
	≥12 months	28 (39)	
Previous heart failure, n (%)			0 (0)
Hypertension, n (%)			50 (70)
Diabetes, n (%)			29 (41)
Dyslipidemia, n (%)			66 (93)
**Smoking, n (%)**			
	Current smoker	14 (20)	
	Former smoker	32 (45)	
	Nonsmoker	21 (30)	
	Unknown	4 (6)	
History of stroke, n (%)			3 (4)
Chronic kidney disease, n (%)			3 (4)
**Echocardiography**			
	LVEF^b^ (%)	57.1 (8.2)	
	LAVI^c^ (mL/m^2^)	37.0 (29.3)	
**Laboratory test**			
	Creatinine (mg/dL)	0.9 (0.3)	
	Total cholesterol (mg/dL)	150.6 (42.3)	
	LDL^d^ (mg/dL)	89.7 (28.6)	
	ProBNP^e^ (pg/L)	370.3 (505.3)	
	Troponin I (ng/mL)	8.8 (14.8)	
	CK-MB^f^ (mg/dL)	20.7 (39.5)	
**Discharge medication**			
	Aspirin	61 (86)	
	P2Y12 inhibitor	37 (52)	
	β-blocker	56 (79)	
	RAS inhibitor^g^	23 (32)	
	Calcium channel blocker	18 (25)	
	Statin	71 (100)	

^a^STEMI: ST-segment elevation myocardial infarction.

^b^LVEF: left ventricular ejection fraction.

^c^LAVI: left atrial volume index.

^d^LDL: low-density lipoprotein.

^e^ProBNP: pro–B-type natriuretic peptide.

^f^CK-MB: creatine kinase-MB.

^g^RAS inhibitor: renin-angiotensin system inhibitor.

### Clinical Outcomes

**Figure 2:**
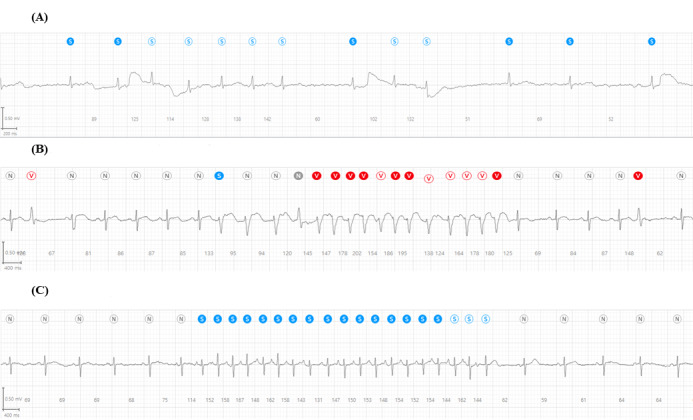
Examples of the detected arrhythmia (A) Atrial fibrillation. (B) Nonsustained ventricular tachycardia. (C) Atrial tachycardia.

The incidence of arrhythmias as detected by ATP-C120 and confirmed by 2 cardiologists (C1 and C2) is shown in Table 2. One participant was concordantly diagnosed with atrial fibrillation by both cardiologists (Figure 2A). The cardiologists determined that atrial premature beats occurred in 65 (92%) (C1) and 60 (85%) patients (C2), and ventricular premature beats occurred in 38 (54%) (C1) and 44 (62%) patients (C2). No ventricular fibrillation was recorded and only 1 nonsustained ventricular tachycardia event was noted by both cardiologists (Figure 2B). Remarkably, 40 (56%) patients (according to both C1 and C2) showed paroxysmal atrial tachycardia, which was sustained less than 2 minutes (Figure 2C).

In Table 3, additional analyses of patients with atrial tachycardia are shown.

In general, there were no significantly different characteristics between patients with atrial tachycardia and those without atrial tachycardia. The timing of monitoring after acute myocardial infarction was not significantly different (55% vs 45%; *P*=.56). The LVEF (mean 56.1%, SD 8.6% vs mean 57.9%, SD 7.8%; *P*=.35) and the size of left atrium or left atrial volume index (mean 32.3, SD 8.9 vs mean 35.6, SD 12.9; *P*=.31) were not significant contributors to atrial tachycardia. However, among medications, the use of β-blockers made a significant difference (70% vs 90%; *P*=.04) (Table 3). Furthermore, when we analyzed participants based on different β-blocker dosages, there were no significant differences (Table 4).

**Figure 3:**
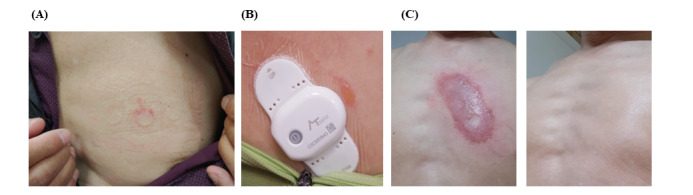
Adverse events from use of ATP-C120 patch.

Lastly, reported adverse events associated with the ATP-C120 patch are described in Table 5. Among the participants, 21 (30%) complained of itching during the patch monitoring period, 2 (3%) experienced abrasion (Figure 3A), 1 experienced bullae (Figure 3B), and 1 showed erosion but fully recovered without scarring or altered pigmentation (Figure 3C).

**Table 2:** Incidence of arrhythmias during follow-up.

Incidence of arrhythmias	Participants (N=71)	
	Cardiologist 1, n (%)	Cardiologist 2, n (%)
Atrial premature beats	65 (92)	60 (85)
Ventricular premature beats	38 (54)	44 (62)
Atrial tachycardia	40 (56)	40 (56)
Nonsustained ventricular tachycardia	1 (1)	2 (3)
Ventricular fibrillation	0 (0)	0 (0)
Atrial fibrillation	1 (1)	1 (1)

**Table 3:** Characteristics of patients with atrial tachycardia (N=71).

Characteristics		Atrial tachycardia		
		Yes (n=40)	No (n=31)	*P* value
Age (years), mean (SD)		68.7 (7.7)	66.2 (8.9)	.22
Male, n (%)		32 (80)	27 (87)	.65
BMI (kg/m^2^), mean (SD)		24.2 (2.9)	24.4 (3.1)	.73
Systolic blood pressure (mm Hg), mean (SD)		128.2 (18.2)	131.8 (19.2)	.43
Diastolic blood pressure (mm Hg), mean (SD)		73.2 (11.3)	71.5 (16.5)	.64
Heart rate (beats/min), mean (SD)		73.2 (11.3)	71.8 (11.2)	.93
Hypertension, n (%)		28 (70)	22 (71)	>.99
Diabetes mellitus, n (%)		18 (45)	11 (35)	.57
Dyslipidemia, n (%)		38 (95)	28 (90)	.65
**Smoking, n (%)**				.74
	Current smoker	7 (19)	7 (23)	
	Former smoker	17 (46)	15 (50)	
	Nonsmoker	13 (35)	8 (27)	
History of stroke, n (%)		0 (0)	3 (10)	.08
Chronic kidney disease, n (%)		3 (8)	0 (0)	.25
Diagnosis of STEMI^a^, n (%)		7 (23)	16 (40)	.19
**Postmyocardial infarction** **period, n (%)**				.92
	<6 months	22 (55)	18 (58)	
	6-12 months	2 (5)	1 (3)	
	≥12 months	16 (40)	12 (39)	
**Echocardiography, mean (SD)**				
	LVEF^b^ (%)	56.1 (8.6)	57.9 (7.8)	.35
	LAVI^c^ (mL/m^2^)	32.3 (8.9)	35.6 (12.9)	.31
**Laboratory test, mean (SD)**				
	Creatinine (mg/dL)	1.0 (0.4)	0.9 (0.2)	.34
	Total cholesterol (mg/dL)	148.0 (40.0)	153.8 (45.4)	.58
	LDL^d^ (mg/dL)	87.2 (28.1)	93.3 (29.4)	.41
	ProBNP^e^ (pg/L)	298.1 (316.5)	464.7 (683.0)	.43
	Troponin I (ng/mL)	7.7 (9.7)	10.3 (19.6)	.61
	CK-MB^f^ (mg/dL)	16.0 (20.4)	26.5 (55.0)	.51
**Discharge medications, n (%)**				
	RAS inhibitor^g^	9 (23)	14 (45)	.04
	β-blocker	28 (70)	28 (90)	.04
	Calcium channel blocker	8 (20)	10 (32)	.24
CHA_2_DS_2_-VASc^h^ score, mean (SD)		2.3 (1.2)	2.6 (1.4)	.22

^a^STEMI: ST-segment elevation myocardial infarction.

^b^LVEF: left ventricular ejection fraction.

^c^LAVI: left atrial volume index.

^d^LDL: low-density lipoprotein.

^e^proBNP: pro–B-type natriuretic peptide

^f^CK-MB: creatine kinase-MB.

^g^RAS inhibitor: renin-angiotensin system inhibitor.

^h^CHA_2_DS_2_-VASc: congestive heart failure, hypertension, age ≥75 years, diabetes mellitus, stroke, vascular disease, age 65-74 years, sex category.

**Table 4:** Comparison of incidence of atrial tachycardia with use of different dosages of β-blockers (N=71).

β-blocker	Atrial tachycardia		
	Yes (n=40), n (%)	No (n=31), n (%)	*P* value
No	12 (30)	2 (7)	.03
Low	13 (33)	15 (48)	.27
Intermediate	9 (23)	9 (29)	.73
High	6 (6)	5 (16)	>.99

**Table 5:** Adverse events associated with the ATP-C120 patch.

Reported adverse events	Participants (N=71), n (%)
Itching	21 (30)
Pricking	2 (3)
Abrasion	2 (3)
Erosion	1 (1)
Bullae	1 (1)

## Discussion

### Principal Findings

In this study, we analyzed 71 patients’ ECG signals after myocardial infarction for 11 days. Several studies reported that myocardial infarction may be associated with new-onset arrhythmias. In particular, some fatal arrhythmic events such as ventricular tachyarrhythmia often occur during or immediately after acute myocardial infarction [2,3]. Prolonged conventional ECG monitoring increases the detection rate of arrhythmic events [10]. Therefore, we designed the study to detect arrhythmias in patients with postmyocardial infarction, using the ATP-C120, a new patch device. This device is a single-lead ECG monitoring device that can continuously monitor the ECG signal for up to 11 days. The ATP-C120 has recently demonstrated that its diagnostic capability and safety compares to conventional ECG monitoring systems [11].

In this study, potentially fatal arrhythmias such as ventricular tachyarrhythmias or atrial fibrillation, which can produce devastating events, were scarcely detected. However, a moderate number of participants with postmyocardial infarction had nonsustained atrial tachycardia events. Several studies reported that nonsustained atrial tachycardia and paroxysmal atrial fibrillation share similar electrical stimulating pathways [12]. Other studies reported that episodes of atrial tachycardia may cause the remodeling of the pulmonary vein cardiomyocytes and the left atrium, and that atrial tachycardia is potentially related to arrhythmogenesis of paroxysmal atrial fibrillation [13]. Although in our study the device only detected 1 exact paroxysmal atrial fibrillation, a moderate number of nonsustained atrial tachycardia episodes were recorded, indicating the unstable hemodynamic and electrical impulses of the atria after myocardial infarction.

β-Blocker therapy after myocardial infarction is necessary for survival [14]. Therefore, guidelines based on randomized controlled and large observational studies recommend β-blocker therapy for all patients after myocardial infarction [15,16]. In our study, nonsustained atrial tachycardia was more frequently found in patients who were not receiving β-blocker therapy. The antiarrhythmogenic effect of β-blocker therapy may stabilize atrial electrophysiology. Therefore, this study suggests that long-term β-blocker therapy after myocardial infarction for prevention of atrial degeneration and new-onset atrial fibrillation needs to be further investigated. Interestingly, the dose of β-blocker did not affect the incidence of paroxysmal atrial tachycardia. This implies that β-blockers may be important when used at any tolerable dose after acute myocardial infarction.

### Strengths and Limitations

This study was a comprehensive analysis of the incidence of postmyocardial infarction arrhythmias. One major limitation of our study was the small number of patients. Therefore, only a few clinically important arrhythmic events were recorded. However, this does not indicate that prolonged monitoring after myocardial infarction only shows trivial arrhythmic episodes. This study showed that there was an interestingly high incidence of supraventricular arrhythmic events. This infers the need for further investigation regarding the progression to atrial fibrillation and fatal complications after arrhythmic episodes.

### Conclusions

A wearable ECG monitoring patch device is easy to apply and can detect ECG rhythm disturbances in patients with postmyocardial infarction. Further study is necessary regarding clinical implications and therapeutic approaches for early detected arrhythmias after myocardial infarction to prevent adverse outcomes among patients.

## Abbreviations

ECG: electrocardiogram
LVEF: left ventricular ejection fraction
